# Precision and Safety of an Intravascular Temperature Management System for Postcardiac Arrest Syndrome Patients: A Multicenter Clinical Trial (COOL-ARREST JP)

**DOI:** 10.1089/ther.2019.0046

**Published:** 2020-09-03

**Authors:** Tsuyoshi Maekawa, Kotaro Kaneda, Ryosuke Tsuruta, Yasuhiro Kuroda, Ken Nagao, Hiroshi Rinka, Takeshi Takahashi, Hiroyuki Yokota, Shin-Ichi Shirai, Mamoru Hase, Joji Kotani, Shigeatsu Endo

**Affiliations:** ^1^ Yamaguchi Prefectural University, Yamaguchi, Japan.; ^2^ Advanced Medical Emergency and Critical Care Medicine, Yamaguchi University Hospital, Yamaguchi, Japan.; ^3^ Emergency Medical Center, Kagawa University Hospital, Miki, Japan.; ^4^ Cardiovascular Disease Center, Nihon University Hospital (Surugadai), Nihon University Hospital, Tokyo, Japan.; ^5^ Emergency and Critical Medical Care Center, Osaka City General Hospital, Osaka, Japan.; ^6^ Kumamoto Medical Center, Kumamoto, Japan.; ^7^ Emergency and Critical Care Medicine, Nippon Medical School Hospital, Tokyo, Japan.; ^8^ Cardio-Vascular Center, Kokura Memorial Hospital, Fukuoka, Japan.; ^9^ Department of Traumatology and Critical Care Medicine, Sapporo Medical University Hospital, Sapporo, Japan.; ^10^ Center for Emergency and Critical Care Medicine, Kobe University Hospital, Kobe University Hospital, Kobe, Japan.; ^11^ Advanced Emergency and Critical Care Center, Iwate Medical University Hospital, Morioka, Japan.

**Keywords:** therapeutic hypothermia, intravascular cooling, neurolept-anesthesia, postcardiac arrest syndrome, cooling speed, cerebral performance category, prospective multicenter trial

## Abstract

Rapid induction and maintaining a target temperature of 32.0–36.0°C within a narrow range for <24 hours are essential, but those are very hard to perform in postcardiac arrest syndrome (PCAS) patients. We investigated the usability of an intravascular temperature management (IVTM) system with neurolept-anesthesia (NLA; droperidol and fentanyl). Single-arm, prospective multicenter trial was carried out in the seven university and the three affiliated hospitals. In the 24 comatose PCAS patients, the target temperature (33.0°C) was rapidly induced and maintained for 24 hours using an IVTM system with NLA. The rewarming speed was 0.1°C/h until 36.5°C and was maintained for 24 hours. The primary end point was the ability to achieve ≤34.0°C for <3 hours after starting cooling, and the secondary end points were the cooling rate, deviation from the target temperature, and adverse events. Cerebral Performance Category (CPC) score at 14 days was also evaluated. Statistical analyses were performed by SPSS software, using the intention-to-treat data sets. The target temperature of ≤34.0°C was reached by 45 minutes (35–73 minutes) and was within 3 hours in all patients. The cooling rate from 36.4°C to 33.0°C was 2.7°C/h (2.4–3.6°C/h). The temperature of 33.1°C (33.1–33.1°C) and 36.7°C (36.6–36.9°C) for 24 hours each was held during the maintenance and the after rewarming phases, respectively. Temperature deviations >0.2°C from 33.0°C in the maintenance phase occurred once each in two patients. The favorable neurological outcomes (CPC1, 2) were relatively good (50%). Five patients experienced serious adverse events; none was device related. We rapidly achieved therapeutic hypothermia within a narrow temperature range without major complications using the IVTM system with NLA in PCAS patients.

## Introduction

Targeted temperature management or therapeutic hypothermia (TH) of 32.0–36.0°C is essential for postcardiac arrest syndrome (PCAS) patients to achieve favorable neurological outcomes (Bernard *et al.*, 2002; The Hypothermia After Cardiac Arrest Study Group, 2002; Morrison and Deakin, 2010; Nielsen *et al.*, 2013). There are many methods for TH. Hoedemaekers *et al.* (2007) compared the five methods and determined that an intravascular cooling system achieved the greatest cooling speed and the smallest deviation. There is a clear clinical need to introduce a cooling method capable of cooling patients more rapidly and maintaining the target temperature more tightly than existing methods.

In this study, we evaluated the safety and effective use of an intravascular temperature management (IVTM) system together with an appropriate anesthetic method in PCAS patients.

## Materials and Methods

### Study population

We conducted a nonblinded, single-arm, prospective multicenter (10 hospitals) clinical trial in Japan between May 2013 and January 2014. The protocol was approved by the Institutional Review Boards at all participating hospitals, and the trial was registered on the U.S. National Institute of Health ClinicalTrials.gov website (identifier: NCT01847482). The trial was overseen by an independent data safety monitoring board.

The inclusion criteria were in-hospital or out-of-hospital cardiac arrest with ventricular tachycardia/fibrillation (VT/VF) or witnessed cardiac arrest with pulseless electrical activity/asystole within 15 minutes of onset; inability to follow verbal instructions; age 20–80 years; and the ability to begin cooling <6 hours after return of spontaneous circulation (ROSC).

Exclusion criteria were traumatic cardiac arrest; patient temperature <35.0°C upon admission; pregnancy; terminal disease; severe hemorrhage; unstable arterial blood pressure with catecholamine support; hypersensitivity to heparin; systemic infection or sepsis/septic shock; thrombocyte count <30,000/mm^3^; severe hepatic, renal, or cardiac failure; contraindication for femoral vein access; using percutaneous cardiopulmonary support; continuous hemodiafiltration; or if the physician determined the patient to be ineligible for the study. Eligible patients were registered in the study after we obtained informed consent from the next of kin.

### Anesthesia and muscle relaxant

Neurolept-anesthesia (NLA) comprised droperidol [neuroleptic effect, initial: 0.5 mg/kg, divided by 1/10–1/5 doses with fluid loading to keep arterial pressure, because of its alpha 1 blocking effect; maintenance: 0.025 mg/(kg·h)] and fentanyl [narcotic and analgesic effects, initial: 0.01 mg/kg; maintenance: 1.0 μg/(kg·h)] (Atkinson *et al.*, 1977; Maekawa *et al.*, 2015). Supplemental midazolam was used, when the physician decided to need the patients unconscious in the present study. During the induction and the maintenance phases, patients were intravenously administered a muscle relaxant, either pancuronium or vecuronium [0.10 mg/kg, 0.05 mg/(kg·h)]. During the rewarming and the normal temperature maintaining phases, patients were administered the same muscle relaxant as deemed necessary.

### Study intervention

Patients were intubated and manually or mechanically ventilated. Percutaneous coronary artery intervention was also performed if necessary. Up to 30 mL/kg of cold (4°C) dextrose-free crystalloid solution or plasma expander could be transfused until the start of the IVTM cooling system (Bernard *et al.*, 2003; Kliegel *et al.*, 2005). The IVTM system comprised a Thermogard XP^®^ (TGXP) and Start-up Kit fitted with a Quattro^®^ or an ICY^®^ (4 or 3 balloons, used depending upon his/her high in 17 or 7 patients) intravascular heat exchange catheter (ZOLL Circulation, San Jose, CA) was used under NLA with neuro-oriented intensive care (Atkinson *et al.*, 1977; Maekawa *et al.*, 1997, 2015; Polderman and Herold, 2009). The intravascular heat exchange catheter was introduced into the inferior vena cava through a femoral vein using the Seldinger technique and was connected to the circuit. The tip of the catheter in his/her inferior vena cava was ensured by a simple X-ray film. Deep vein thrombosis in their inferior vena cava was evaluated by an echo angiography, a computed tomography, and/or blood examination (platelet count, fibrinogen, fibrinogen degeneration product, and D-dimer).

The primary site for temperature measurement was the middle of the esophagus, and the secondary site was the bladder. The probe and the circuit were connected to the TGXP, and the esophageal probe provided thermal feedback. Temperature was recorded at 1-minute intervals using the IVTM device and every 20 minutes manually. A total of 500 mL saline was circulated through the balloons. Patients were cooled as quick as possible to the target temperature of 33°C with a permitted range of 32.0–34.0°C for 24 hours during the maintenance phase, and body temperature was maintained at 36.5°C for 24 hours after the rewarming phase. The rewarming speed was controlled at 0.1°C/h.

### Evaluation

The primary end point was the percentage of patients whose temperature reached ≤34.0°C within 3 hours using the IVTM cooling system. Secondary end points were as follows: deviation of body temperature from the target temperature (33.0°C) during the use of the IVTM system; cooling rate (change in temperature per unit of time); and safety measures, which included adverse events, abnormal changes in laboratory test values, and IVTM system malfunctions during the study. The mean error was also calculated from the difference between the recorded temperature and the target temperatures to quantify temperature deviation (Matthews *et al.*, 1990). Cerebral Performance Category (CPC) score was also evaluated 14 days after ROSC (Safar, 1981).

### Statistical analysis

All data were analyzed descriptively using the intention-to-treat approach. Data are expressed as the median (interquartile range) for continuous variables or as the number of patients (percent) for categorical variables. All analyses were performed using SPSS software version 22.0 (IBM Corp., Armonk, NY).

## Results

**FIG. 1.**
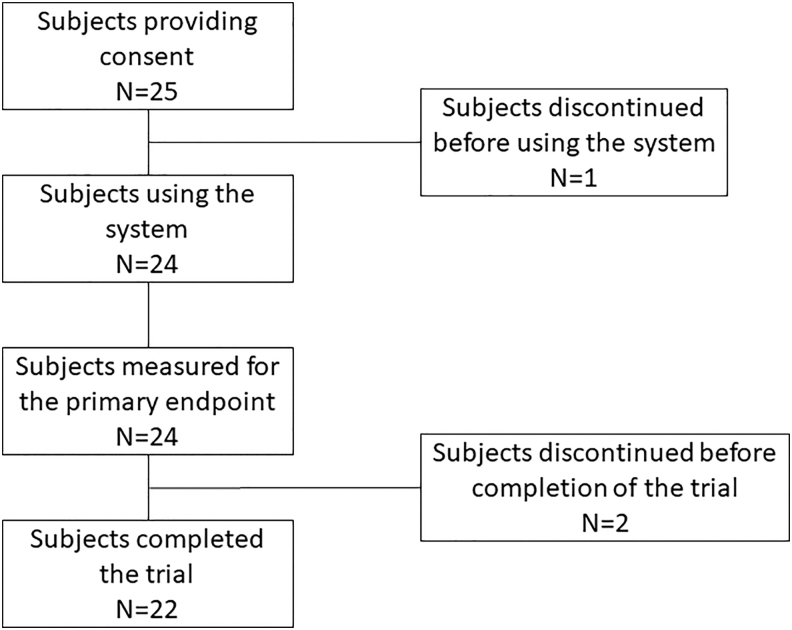
Patient disposition.

**FIG. 2.**
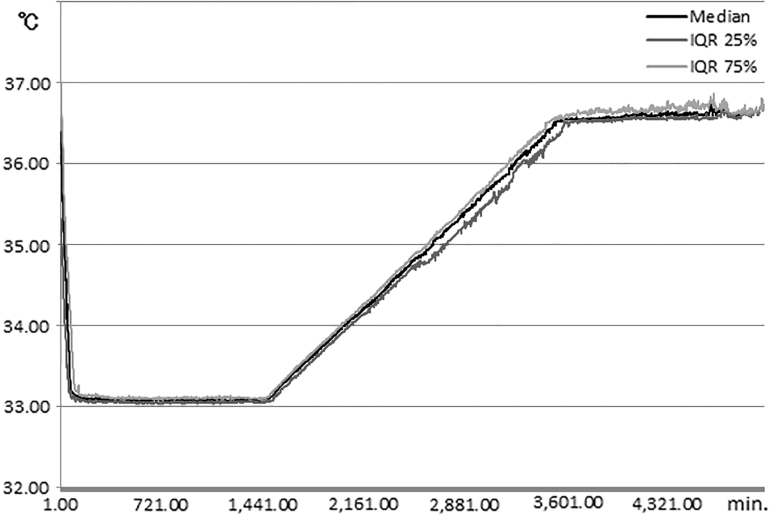
Core body temperature measured every minute in 19 patients who completed the protocol and whose temperature was recorded on the device. The values are expressed as the median and interquartile range.

All of 25 comatose PCAS patients, who experienced out-of-hospital cardiac arrest, were initially enrolled (Fig. 1). Among 24 IVTM system used patients, 2 patients were withdrawn after the target temperature of 33°C was reached, because of the physicians' decision. The data obtained for these two patients during and after the maintenance phase were excluded from the analyses, except for their neurological outcomes. Patient characteristics, physiological variables, and laboratory data on admission or upon stabilization are shown in Table 1. The median volume of cold fluid transfusion until the start of the IVTM cooling system was 21 mL/kg (14–27 mL/kg), and the core body temperature was 36.4°C (35.2–36.8°C) at that time. The time from ROSC to the start of cooling was 249 minutes (216–342 minutes) (Table 2). The time to reach the target temperature of ≤34.0°C was 45 minutes (35–73 minutes) (Table 3). The longest time was 180 minutes, so the primary end point was achieved in all 24 patients. The cooling rate during the induction phase was 2.7°C/h (2.4–3.6°C/h). The patient temperature was 33.1°C (33.1–33.1°C) at 8 and 24 hours during the maintenance phase, respectively. After rewarming (the target temperature: 36.5°C), it was 36.6°C (36.5–36.6°C) at 8 hours and 36.7°C (36.6–36.9°C) at 24 hours (Table 3). Manual temperature recordings at 20-minute intervals showed that the recorded temperature differed from the target temperature by ≥0.2°C in two patients, once in each patient, but there was no deviation of ≥0.5°C during the maintenance phase. Data recorded minute by minute were available for 19 patients (Fig. 2). Five patients were not included in this figure, because two patients were withdrawn, data of two other patients were incomplete, and data of the other patient were overwritten by data for the next patient. The Figure 2 confirms that the patients' temperature is tightly controlled by the IVTM system in each of the temperature management phases. The mean error was 0.09°C (0.06–0.11°C) during the maintenance phase (targeted 33.0°C) and 0.11°C (0.07–0.17°C) after rewarming (targeted 36.5°C).

**Table 1.** Patient Characteristics on Admission and Physiological and Laboratory Variables Following Stabilization

Patient characteristics on admission		Physiological and laboratory variables	
Age (years)	60 (43–70)	Mean BP (mmHg)	98 (64–123)
Male	21 (87.5%)	HR (bpm)	110 (74–123)
Height (cm)	170 (162–174)	PaO_2_ (mmHg)	128 (64–316)
Weight (kg)	70 (60–75)	PaCO_2_ (mmHg)	47.9 (37.1–53.3)
BMI (kg/m^2^)	24.4 (23.2–25.3)	pH	7.25 (7.18–7.30)
BSA (m^2^)	1.75 (1.63–1.85)	BE (mEq/L)	−8.4 (−11.5 to −5.1)
Characteristics of cardiac arrest on admission		Lactate (mg/dL)	50.4 (32.0–70.0)
Out of hospital	24 (100%)	Glucose (mg/dL)	244 (200–323)
Witnessed	21 (87.5%)	CRP (mg/dL)	0.11 (0.09–0.22)
VT·VF	18 (75.0%)	GCS on admission	3 (3–6)
PEA	6 (25.0%)		

Values are expressed as the median (interquartile range) or *n* (%) for the intention to treat population (*n* = 24).

BE, base excess; BMI, body mass index; BSA, body surface area; BP, blood pressure; CRP, C-reactive protein; GCS, Glasgow coma scale score; HR, heart rate; PaCO_2_, partial pressure of arterial carbon dioxide; PaO_2_, partial pressure of arterial oxygen; PEA, pulseless electrical activity; VF, ventricular fibrillation; VT, ventricular tachycardia.

**Table 2.** Duration from Cardiac Arrest or Return of Spontaneous Circulation and Volume of Cold Fluid Transfusion Before the Start of the Intravascular Temperature Management Cooling System

Clinical variables	
Cardiac arrest to admission (minutes)	32 (26–120)
Cardiac arrest to ROSC (minutes)	20 (14–35)
Cardiac arrest to start cooling (minutes)	274 (242–359)
ROSC to start cooling (minutes)	249 (216–342)
Volume of cold fluid transfusion (mL/kg)^a^	21 (14–27)

Values are expressed as the median (interquartile range) for the intention-to-treat population (*n* = 24).

^a^ Cold fluid (4°C, up to 30 mL/kg body weight) could be transfused until the start of the IVTM cooling system.

IVTM, intravascular temperature management; ROSC, return of spontaneous circulation.

**Table 3.** Core Body Temperatures and Times to Reach Target Temperatures

Timing of core body temperature record	Patient number	°C
At registration	24	36.1 (35.6–37.0)
Just before cooling	24	36.4 (35.2–36.8)
Start of the maintenance phase	24	34.0 (33.9–34.0)
8 Hours after reaching the target temperature	22	33.1 (33.1–33.1)
24 Hours after reaching the target temperature	22	33.1 (33.1–33.1)
At the time of reaching the normal temperature	22	36.0 (36.0–36.0)
8 Hours after reaching the normal temperature	22	36.6 (36.5–36.6)
24 Hours after reaching the normal temperature	22	36.7 (36.6–36.9)
*Times to reach target temperatures*	*Patient number*	*Minutes or °C/h*
Time from starting cooling to reach 34°C (minutes)	24	45 (35–73)
Rate of cooling to reach 33°C (°C/h)	24	2.7 (2.4–3.6)

Values are expressed as the median (interquartile range).

The values for two patients were excluded after the target temperature of 33°C was reached, because the physicians decided that the patients' conditions were too poor to continue therapeutic hypothermia.

One IVTM system malfunctioned due to a leak in the Start-up Kit line after the rewarming phase. The Start-up Kit line was exchanged, and the treatment was completed without harm to the patient. Serious adverse events occurred in five patients. Two patients were withdrawn from the study protocol during the maintenance phase due to recurrent VF, which necessitated rewarming, as described above. The other patient had VF due to hypokalemia, but a normal rhythm was achieved using standard treatments. One patient had acute respiratory distress syndrome on 11th day after ROSC, and it was improved by ordinary treatments. There was a report of deep vein thrombosis around the IVTM heat exchange catheter in one patient and treated without any other complication.

CPC at 14 days were favorable, with CPC grades of 1, 2, 3, 4, and 5 in 11, 1, 2, 9, and 1 patient in the present study, respectively, while those were in 2, 0, 1, 3, and 0 in the pulseless electrical activity patients, respectively.

## Discussion

In the present study, all of the patients were cooled quickly to 34.0°C within 3 hours, actually it was 45 minutes (35–73 minutes) after starting the cooling process, and the cooling rate from 36.4°C to 33.0°C was 2.7°C/h (2.4–3.6°C/h). The target core body temperature (33.0°C) was maintained within an extremely narrow range, and patients were rewarmed smoothly (Table 3 and Fig. 2). These results provide further evidence for the precision and usability of the IVTM system, combined with NLA and neuro-oriented intensive care (Atkinson *et al.*, 1977; Maekawa *et al.*, 1997, 2015; Polderman and Herold, 2009).

### Cooling speed and the rate to reach the targeted temperature

The target temperature of 34°C was achieved in 260 minutes, mainly using surface cooling by Nielsen *et al.* (2013), in 188 and 170 minutes using an IVTM system and ordinary surface cooling by Tømte *et al.* (2011), and in 330 minutes using an IVTM system with a two balloon catheter by Deye *et al.* (2015). The target temperature of 33°C was achieved in 190 minutes by Keller *et al.* (2003), in 210 minutes using an IVTM system with a two balloon catheter by Al-Senani *et al.* (2004), in 179 or 208 minutes using an IVTM system (62% of patients) by Kirkegaard *et al.* (2017), and in 89 minutes (42–155 minutes) using the same IVTM system (Thermogard XP) with a three or four balloon catheter by Sawyer *et al.* (2019). Concerning about the cooling rate to reach 33.0°C, it was 0.8°C/h, achieved by Al-Senani *et al.* (2004), and 0.39°C/h by Deye *et al.* (2015), respectively. All these reports took much longer duration and much slower cooling rates than those of ours (Table 3 and Fig. 2). Possible reasons might be their cooling methods; they used surface cooling and/or the endovascular cooling system with a two-balloon catheter, except for Sawyer's trial (2019), while we used an IVTM system with a three or four balloon catheter.

When the patients were limited to the VT/VF, Schock *et al.* (2018) reviewed the report of Howes *et al.* (2010), and rapid cooling to 34.0°C within 3.5 hours of ROSC and cooling rate >3.0°C/h without cold fluid volume load yielded a higher rate of good neurological outcome (around 80%). We calculated the duration from ROSC to 34.0°C in the VT/VF patients (*n* = 18). It was 5.4 hours, and the good neurological outcome (CPC 1 + 2) was 55.6% with similar cooling speed, 3.0°C/h to 34.0°C after the start of cooling. The difference of the neurological outcome would come from our delayed start of cooling.

Sakamoto *et al.* (2014) reported much quicker cooling methods, such as an extracorporeal cardiopulmonary support device, which were compared to conventional cooling methods and had better neurological outcome, 11.2% versus 1.5% at 6 months, respectively. Polderman *et al.* (2015) reported an ultrarapid induction method, an automated peritoneal lavage system using ice-cold fluids, and had much better neurological outcome as 56.4% at 6 months in VT/VF patients on arrival, although their methods were more invasive than our methods.

### Deviation from the targeted temperature

Figure 2 shows the minute-by-minute recording of the core body temperature measured in the middle of esophagus for 19 patients. The mean error was 0.09°C (0.06–0.11°C) during the maintenance phase and 0.11°C (0.07–0.17°C) after the rewarming phase. Considering possible differences in calculations, we demonstrated better temperature management in the present study than was observed in prior studies. The precision and rapid cooling rate were due to direct intravascular blood cooling, in which cooled blood quickly and directly suppressed the function of the thermal control centers in the anterior hypothalamic-preoptic area and the posterior hypothalamus (Guyton and Hall, 2005). By contrast, body surface cooling constricts subcutaneous arteries and arterioles, which might disturb thermal exchange and stimulate the thermal control centers using cutaneous thermal receptors, ultimately triggering shivering (Guyton and Hall, 2005). These physiological responses may enormously influence the cooling speed and temperature precision.

### Anesthesia and muscle relaxants

TH itself might be seriously harmful stimuli, so that appropriate anesthesia or sedation with analgesics must be needed, as Canadian TH guideline strongly suggested (Howes *et al.*, 2016). Propofol and midazolam are widely used as sedatives, and fentanyl or morphine is often used as analgesics (Chamorro *et al.*, 2010). However, those sedatives decreased cerebral blood flow (Oshima *et al.*, 2002; Kaisti *et al.*, 2003; Ogawa *et al.*, 2010; Reves *et al.*, 2010), while NLA which was used in the present study did not decrease cerebral blood flow in humans (Sari *et al.*, 1972; Patel and Drummond, 2010). Besides its sedative and amnesic effects, droperidol dilated peripheral arteries and arterioles by blocking alpha 1 adrenoceptors and improved peripheral circulation (Atkinson *et al.*, 1977) and facilitated heat exchange throughout the body, while we administered dobutamine (beta agonist) to maintain cardiac output, MAP (60–150 mmHg), and cerebral blood flow, as deemed necessary. Narcotic properties of fentanyl partly suppressed shivering, suppressed endogenous catecholamine release, and prevented vasoconstriction (Fukuda, 2010).

Nondepolarizing muscle relaxants which we used completely block neuromuscular junctions, thus suppressing shivering and preventing an increase in body temperature (Naguib and Lien, 2010). In PCAS patients, Salciccioli *et al.* (2013) reported that continuous neuromuscular blockade improved the survival rate. Therefore, our methods of anesthesia and muscle relaxation would contribute good temperature management for PCAS patients.

### Neurological outcomes

Overall, 12/24 patients (50%) had a favorable neurological outcome, 14 days after ROSC, even though TH had started relatively late, 249 minutes (216–342 minutes) after ROSC (Table 2). The reasons might be included, that is, the high rate of witnessed PCAS patients (87.5%), the relatively short resuscitation period (20 and 14–35 minutes), the prevention of pyrexia by cold fluid transfusion, and tight management of the body temperature for >72 hours (Tables 2, 3, and Fig. 2).

### Limitation

There are limitations of our clinical study. First, because it was a nonblinded, single-arm clinical trial, the results cannot be directly compared to those reported for other cooling methods. Second, this was a government-required clinical trial for regulatory approval of the IVTM system in Japan and, as such, it was financially and logistically supported by the manufacturer. Therefore, safety was the most important factor. In the future, it would be beneficial to perform multicenter randomized controlled clinical trials using the IVTM system with NLA to treat PCAS patients.

In conclusion, the IVTM cooling system with NLA successfully and rapidly induced TH with low deviation from the targeted temperature and without major complications in PCAS patients. The results indicate that the IVTM system is suitable for managing TH and rewarming in PCAS patients.

## Figures and Tables

**Table:** 

**Sapporo Medical University Hospital** Chief Investigator: Mamoru Hase Sub Investigator: Katsutoshi Tanno, Noriaki Kokubun, Shuji Uemura, Junichi Nishida, Hisashi Fumiya, Eiji Sakawaki
**Iwate Medical University Hospital** Chief Investigator: Shigeatsu Endo Sub Investigator: Katsutoshi Terui, Tomohiro Takahashi, Chikahiko Hida, Chiaki Onodera
**Nippon Medical School Hospital** Chief Investigator: Hiroyuki Yokota Sub Investigator: Akira Fuse, Tomohiko Masuno, Hideyoshi Onda, Hironori Ishii, Masato Miyauchi, Fumihide Kin, Atsuko Tsujii, Takeshi Suzuki, Masanori Arai, Jun Hagiwara, Takeshi Tsukamoto, Keiji Tanaka, Koichi Akutsu, Takeshi Yamamoto, Yusuke Hosokawa, Hiroshige Murata, Mitsunobu Kitamura, Hideki Miyaji, Hidekazu Kawanaka, Tetsuhiro Hashizume, Shoji Yokobori
**Osaka City General Hospital** Chief Investigator:Hiroshi Rinka Sub Investigator: Isanori Miyaichi, Hideki Arimoto, Akihiro Fuke, Junichi Ishikawa, Takaya Morooka, Daisuke Arashi, Yuko Hamanaka, Ken Morimoto, Junpei Okawa, Taneaki Shigemitsu
**Kagawa University Hospital** Chief Investigator: Yasuhiro Kuroda Sub Investigator: Kenya Kawakita, Yuko Abe, Natsuyo Shinohara, Shuichi Okubo, Kazutaka Kiritsume, Hideyuki Hamatani, Koshiro Takano
**Yamaguchi University Hospital** Chief Investigator: Ryosuke Tsuruta Sub Investigator: Kotaro Kaneda, Yasutaka Oda, Yoshikatsu Kawamura, Motoki Fujita, Takashi Nakahara, Yasutaka Koga, Yasuaki Ogino, Shinya Fukuda, Ichiko Mizuguchi, Mari Otsuji, Takefumi Yagi, Takashi Miyauchi, Ryo Tanaka, Masaki Toya, Tadashi Kaneko
**Kokura Memorial Hospital** Chief Investigator: Masashi Iwabuchi, Kenji Ando Sub Investigator: Shinichi Shirai, Junichi Shimotakahara,
**National Hospital Organization, Kumamoto Medical Center** Chief Investigator: Takeshi Takahashi Sub Investigator: Masahiro Harada, Toshihiro Sakurai, Maki Kitada, Shu Yamada, Kohei Karino, Tadashi Era
**Nihon University Hospital** Chief Investigator: Ken Nagao Sub Investigator: Kimio Kikushima, Kazuhiro Watanabe, Shingo Furuya, Kiyoshi Iida, Mitsunobu Enomoto, Masaaki Matsumoto, Takeshi Soga, Harumi Hirose, Masakazu Matsuzaki, Tsutomu Kobari, Koichiro Kazeto, Kei Nishikawa
**Center for Emergency and Critical Care Medicine, Kobe University Hospital, Emergency and Critical Care Center, Hyogo College of Medicine Hospital** Chief Investigator: Joji Kotani **Hyogo College of Medicine Hospital** Sub Investigators: Atsunori Hashimoto, Takahiro Ueda, Takaaki Osako, Taihei Yamada, Kenichi Matsuda, Norichika Yoshie, Hiroyuki Sakata, Sana Okamoto, Toru Arii, Hideyuki Kijima, Kojiro Miki, Kenichi Fujii, Ken Kotani, Tadashi Fukunaga, Takahiro Imanaka, Tomotaka Ando, Hiroto Tamaru
**Yamaguchi Prefectural University** Chief Investigator: Tsuyoshi Maekawa.

## Authors' Information

COOL-ARREST JP Clinical Trial Group:

## Declarations

### Availability of data and material

The protocol was registered on the U.S. National Institute of Health ClinicalTrials.gov website (identifier: NCT01847482).
